# Technology of Producing Petroleum Coking Additives to Replace Coking Coal

**DOI:** 10.1021/acsomega.1c04075

**Published:** 2021-12-14

**Authors:** Alexey V. Kameshkov, Viacheslav A. Rudko, Renat R. Gabdulkhakov, Maxim Yu. Nazarenko, Maxim K. Starkov, Vladimir G. Povarov, Igor N. Pyagay

**Affiliations:** † Saint Petersburg Mining University, St. Petersburg 199106, Russia; ‡ Ltd Kinef, Leningrad Region, Kirishi 187110, Russia

## Abstract

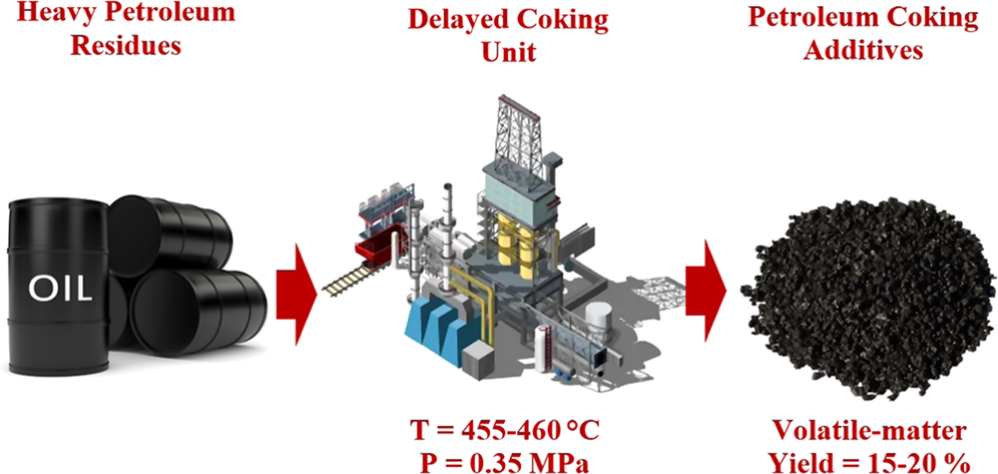

Coke chemical companies often have a deficit of coals of particularly valuable grades, the coking coals. This work studies the opportunity of producing petroleum coking additives using delayed coking during heavy petroleum residue processing. Experiments for the production of a carbon material were conducted using three kinds of heavy petroleum residues of the oil refinery plant Ltd Kinef: the vacuum residue from crude atmospheric and vacuum distillation units (VR1), the vacuum residue from the vacuum distillation hydrocracking unit (VR2), and the visbreaker residue from the visbreaking unit (VR3). For the produced carbon material, the quality indicators were determined, and X-ray diffraction, thermogravimetric, and differential thermal analyses were conducted. The petroleum coking additive produced instead of the typical petroleum coke under a milder temperature regime had the required quality indicators, particularly, the volatile-matter yield within the range from 15 to 25 wt %, to be used in metallurgical production for partial replacement of coking coals in the charge to produce metallurgical coke.

## 1 Introduction

Analysis of the results presented in studies^1−3^ provides an insight that in the future, the replacement of most hydrocarbon resources with alternatives is impossible, so feedstock resources will be relevant for their use in processing. The most in-demand thermal processing of heavy petroleum residues in the world is delayed coking; this process makes it possible to extent petroleum processing to produce commodities at the oil refinery plant up to 98%.^4,5^ In 2021, the total crude material capacity of the delayed coking units in Russia was about 13.6 million tons.^6,7^ Using this process, the light petroleum product output can be increased, as well as the commodity assortment can be expanded with carbon materials.^8−10^ As a production alternative for conventional coarse petroleum coke at delayed coking units, production of petroleum coking additives is suggested.^11,12^

Petroleum coke, which is a potential replacement for coking coals in the production of metallurgical coke, has received the name of a petroleum coking additive in Russia and the CIS countries. A petroleum coking additive is a carbon bottom product of a delayed coking unit at petroleum refineries, which is obtained under a “softer” thermal regime (455–475 °C) than petroleum coke (495–505 °C). The distinctive properties of the petroleum coking additive are a high volatile-matter yield, from 15 to 25 wt % (relative to petroleum coke, usually up to 9–12 wt %).^9,13,14^ In addition, the petroleum coking additive, in contrast to petroleum coke used as a raw material for the production of electrodes or anodes, does not have such strict restrictions on the sulfur content (up to 4.8 wt %). This makes it possible to consider low-grade sulfur and high-sulfur heavy oil residues from hydrocarbon processing as cheap feedstock for delayed coking units. Table 1 shows the requirements for quality indicators of the petroleum coking additive (TU 0258-229-0019437-2008) and, for comparison, the requirements of electrode (TU 38.301-19-99-99) and needle (SuperPremium grade) petroleum cokes are provided.

**Table 1:** Main Technical Requirements for Petroleum Coking Additives and Their Comparison with the Requirements for Electrode and Needle Cokes

parameters	petroleum coking additive	petroleum electrode coke	petroleum needle coke
(1) moisture content (*W*^a^), %, no more	10.0	3.0	
(2) ash content (*A*^a^), %, no more	2.00	0.6–0.8	0.4
(3) sulfur content, wt %, no more	4.8	1.5–1.7	0.5
(4) volatile-matter yield (*V*^daf^), %, within (no more)	15.0–25.0	12.0	

Irrespective of the fact that Russia takes the third place in the world in coking coal production (more than 80.0 million tons per annum) after China and Australia, coke chemical companies suffer from a shortage of coals of particularly valuable grades: coking (K), coking lean (KO), and lean caking coal (OS).^15−17^ It should be noted that the demand for coking coals of these grades will persist from a long-term perspective, as the main consumer of coal coke, the blast-furnace iron-making, is still the main cast-iron and steel making process in the world.^18−20^ For example, the main consumers of the coal coke produced in Kuznetsk, Pechorsk, and Yuzhno-Yakutsk fields in the Russian market^21^ are the following large metallurgical and coke chemical companies: Altay Coke Chemical Plant, West-Siberian Metal Plant, Kemerovo Coke Chemical Plant, Novolipetsk Steel Plant, Chelyabinsk Metallurgical Plant, and others.^15^ The deficit of K, KO, and OS coals, as well as coking fat (KZh) and fat coals (Zh), is compensated for by adding into the charge the other coal grades whose reserves are much more abundant: gas coal (G), gas fat lean coal (GZhO), sinter low-caking low-metamorphized coal (KSN), sinter low-caking coal (KS), long flame coal (DG), lean caking coal (TS), and low-caking coal (SS) that are second to coking coals in quality and degrade the charge, which finally affects the coke quality.^15,16^

In general, the Gray–King coke method is used for the determination of the caking power.^22^ This indicator characterizes the caking power of coal by the type and characteristics of the nonvolatile residue (ISO 502:2015).^23^ In addition, the free-swelling index (FSI) is also used, which is a measure of the increase in the coal volume when heated under certain conditions (ASTM D-720; ISO 335).^24,25^ The main physical and chemical properties of the different coal grades by the Russian classification according to GOST 25543-2013 “Brown coals, hard coals and anthracites. Classification according to genetic and technological parameters” with the appropriate FSI^26^ are given in Table 2.

**Table 2:** Physical and Chemical Properties of Coals According to GOST 25543-2013

rank		volatile-matter yield, %	plastometric index, mm	FSI
gas fat lean coal	GZhO	<38.0	10–16	6
gas fat coal	1GZh	>38.0	>16	71/2
	2GZh	>36.0	17–25	
fat coal	1Zh	28.0–35.9	14–17	81/2
	2Zh	>36	>26	
coking fat coal	KZh	25.0–30.0	18	8
sinter coal	Κ	24.0–24.9	13–17	5–51/2
coking semilean coal	ΚO	24.0–24.9	10–12	4
forge coal	OS	<21.9	6–12	3
lean coking coal	TS	<22.0	<6	1
low-coking coal	SS	28.0–35.9	<6	1
lean coal	T	8.0–15.9		1

To produce the high-quality coal coke, the charge should meet the following requirements of main indicators: the ash content should not exceed 8–10%, the volatile-matter yield should be within 26–30%, the sulfur content should not be more than 0.5–1.0%, and the coal plastometric index should not be less than 15–17 mm.^15^

The lack of required valuable coal grades in the market at a favorable price makes the coke chemical companies look for an acceptable replacement. The coking additive produced from crude petroleum can be such a partial replacement in charge production.^14,27^

## 2 Experimental Section

### 2.1 Objects

**Figure 1:**
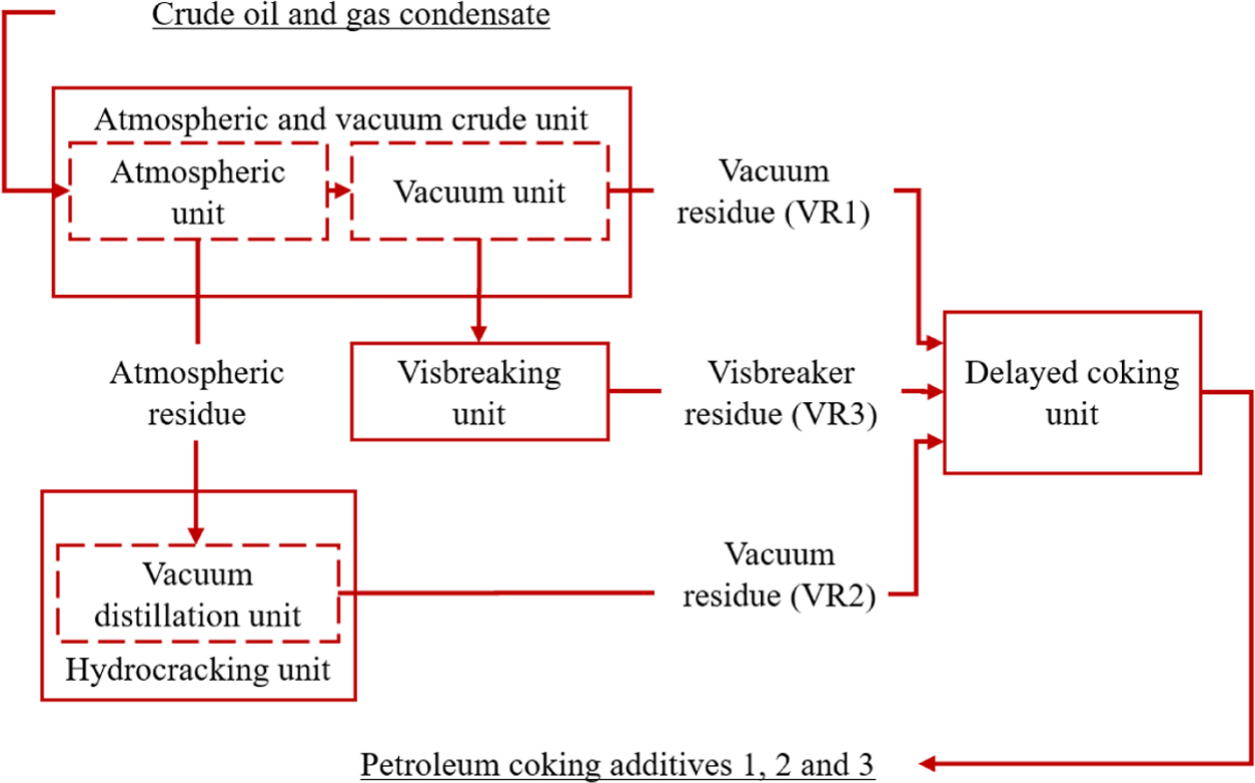
Process flow chart for the petroleum coking additive at Ltd Kinef.

The initial material for the petroleum coking additive was the commercially produced petroleum products at the oil refinery plant Ltd Kinef (Russia).^28^ The plant capacity and process lines are organized so that the potential crude material for the petroleum coking additive industrial production can be three kinds of heavy petroleum residues of the oil refinery plant Ltd Kinef: the vacuum residue from crude atmospheric and vacuum distillation units (VR1), the vacuum residue from the vacuum distillation hydrocracking unit (VR2), and the visbreaker residue from the visbreaking unit (mild thermal cracking) (VR3). In 2023, the plant plans to commission a delayed coking unit where the mentioned heavy petroleum residues can be used for producing the petroleum coking additive as the crude material according to the process flow chart shown in Figure 1.

The quality indicators of the initial material for the petroleum coking additive are given in Table 3.

**Table 3:** Quality Indicators of Heavy Petroleum Residues

quality indicators	VR1	VR2	VR3	test method
density at 15 °C, kg/m^3^	1012.2	1020.5	1032.0	ISO 12185
kinematic viscosity, mm^2^/s				ISO 3104
100 °C	438.2		114.0	
135 °C		195.4		
sulfur, %	2.81	2.95	3.15	ISO 8754
flash point PMCC, °C	308	352	356	ISO 2719
carbon residue—micro method, mass %	20.5	20.8	22.6	ISO 10370
depth of needle penetration at 25 °C, 0.1 mm	450	348		GOST 11501
SARA, wt %				SARA analysis
saturates	8.5	9.2	2.3	
aromatic	52.4	56.9	51.8	
resins	18.7	11.8	20.3	
asphaltenes	20.4	22.1	25.6	

The fundamental difference of the crude material is that VR2 is heavier than VR1 and more prone to coke formation (aromatic hydrocarbon and asphaltene content). In addition, VR1 and VR2 are the products of physical vacuum separation of crude petroleum; as a consequence, they contain no products of thermal decomposition, like in VR3. In this regard, the latter type of raw material has a higher tendency to coke formation. The sulfur content increases when the crude material gets heavier from 2.81 to 3.15%, as most hetero-organic compounds contain high-boiling fractions and asphaltenes.

### 2.2 Methods

#### 2.2.1 Delayed Coking Method

**Figure 2:**
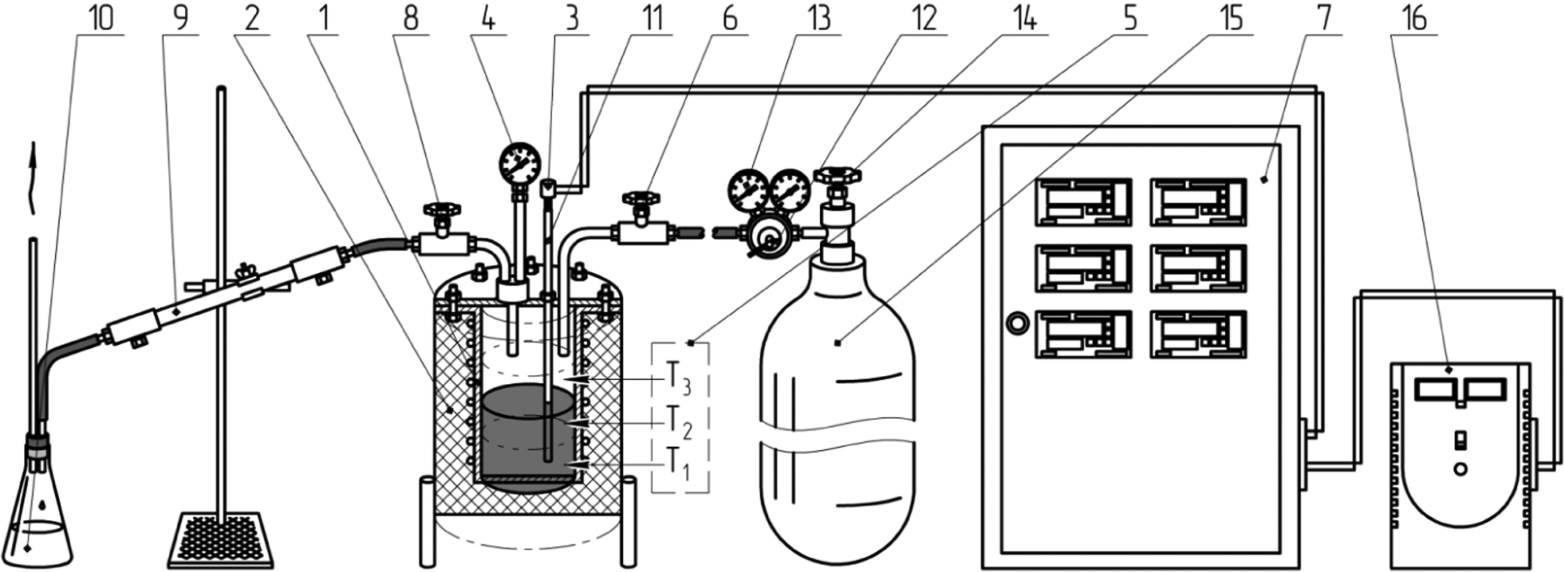
Process flow chart of the laboratory delayed coking unit “UZK-1”: 1, coking reactor; 2, heat insulator; 3, thermocouples of the top and bottom layer of the reactor; 4, reactor pressure gauge; 5, three heating zones; 6, pressure test needle valve; 7, electric control unit; 8, reactor needle valve; 9, double-pipe water heat exchanger; 10, distillate receiver; 11, thermocouple pocket; 12, nitrogen reducer; 13, pressure test pressure gauge; 14, pressure test valve; 15, nitrogen cylinder for purging and pressure testing; and 16, voltage stabilizer.

To produce the petroleum coking additive from the heavy petroleum residues, Ltd Kinef used the laboratory delayed coking unit of the Saint Petersburg Mining University consisting of a reaction unit and a distillate collection unit (Figure 2).

The reaction unit consists of a steel coking reactor and an electric furnace with three independent heating zones to maintain the even temperature by the coking layer height; the reactor is equipped with a pressure gauge for pressure monitoring. The gas–fluid product mix is discharged through the pipe inside the reactor cover, via the needle valve, and then flows into the double-pipe water heat exchanger and distillate collecting bottle, and the hydrocarbon gas is discharged to the exhaust system.

The experiments were conducted at a coking temperature from 455 to 465 °C and at a constant excess pressure of 0.35 MPa for each experiment. Loading for raw materials was 0.247–0.254 kg. After switching to the normal coking temperature, the reactor was held in an isothermal mode until the formation of the gas–fluid products stopped, and, as a result, the pressure decreased in the reactor. The isothermal mode lasted for 60 min. The normal coking parameters for each experiment are shown in Table 4.

**Table 4:** Process Parameters for Producing the Petroleum Coking Additive

parameters	VR1	VR2	VR3
overpressure, MPa	0.35	0.35	0.35
coking temperature (final temperature), °C	455–465	456–460	455–460
heating time to final temperature, min	360	355	360
average heating rate of the coking layer, °C/min	1.55	1.61	1.58
isothermal time at final temperature, min	60	60	60
mass of raw materials, g	254.0	247.0	252.0

#### 2.2.2 Methods for Determining Quality Indicators

The moisture content of the petroleum coke additive was determined in a dryer according to GOST 27589 “Coke. Method for determination of moisture content in analytical sample” (ISO 687:2010) and GOST 33503 “Solid mineral fuel. Method for determination of moisture content in analytical sample” (ISO 11722:2013, ISO 5068-2:2007). Accelerated testing methods were used for determination of moisture content. A total of 2 g of the sample of the petroleum coke additive with a particle size of 125 μm was dried at a temperature of 105 ± 5 °C. The moisture residue was estimated by the mass loss.

The ash content was determined according to GOST 22692 “Carbon materials. Method for determination of ash”. A total of 2 g of the material sample was burnt in a muffle furnace at 815 ± 10 °C and held at the specified temperature until a constant mass was reached. The ash content was estimated by the mass loss.

The volatile-matter yield was determined by heating 1 g of the sample in a porcelain crucible with an air-tight ground-in lid in a muffle furnace at 815 ± 10 °C for 7 min according to GOST 22898 “Low-sulfur petroleum cokes. Specifications” and GOST R 55660 “Solid mineral fuel” (ISO 562:2010). The volatile-matter yield percentage was estimated by the mass loss of the sample weight, exclusive of moisture.

The real density was determined by weighing the petroleum coke additive sample in air and pycnometric fluid (ethanol) according to GOST 22898 “Low-sulfur petroleum cokes. Specifications” and GOST 10220 “Coke. Methods for determination of density and porosity”.

The microstructure was assessed by comparing with the microstructure control scale according to GOST 26132 “Petroleum and pitch coke. Methods of microstructural assessment” using a μVizo-MET-221 microvisor in reflected linearly polarized light with 90–100× magnification.

The sulfur in the petroleum coke additive samples was determined using an XRF-1800 Shimadzu sequential wavelength-dispersive X-ray fluorescence spectrometer with a 3.6 kW Rh anode X-ray tube. The method consists in the determination of sulfur by the introduction of a standard additive into the sample without preliminary incineration.

The microhardness of the petroleum coke samples was determined according to a procedure based on GOST 9450-79 “Measurements microhardness by diamond instruments indentation” and GOST R 8.748-2011 “Metals and alloys. Measurement of hardness and other characteristics of materials upon instrumental indentation” and provided comparable measurement results for the representative coke samples complying with ISO 14577-1 Annex A (Annex A: materials properties estimated by determination of depth of impression and force of indentation). The method consists in making an impression on the test sample surface under a load applied to the diamond point for a certain period of time, with simultaneous measurement of the depth of impression and force of indentation.^29^

#### 2.2.3 X-ray Structure Analysis

The X-ray diffraction analysis of the petroleum coke additive was performed using an XRD-7000 Shimadzu X-ray diffraction apparatus (Cu Kα-radiation, 2.7 kW) at room temperature according to the Debye–Scherrer method. X-ray exposure was conducted at a long accumulation time of 2 s and at a step angle of 0.02°. The nonsymmetric reflections of petroleum cokes were split into peaks whose profile is described by Gaussian with the maximum of 2θ angles characterizing certain structural components of samples.

For a detailed assessment of the thin structure of the petroleum coke additive by the X-ray diffraction method, the interplane distance by diffraction maximum values (002) and (110) and the coherent scattering area in the directions of axes “c” (average crystallites height *L*_c_) and “a” (average hexagonal layer diameter *L*_a_) were used in this work. To determine the interplane distance (*d*_002_ and *d*_110_) in Å petroleum coke additive samples, the calculation was performed according to Bragg’s law^30,31^
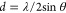
1where λ = 1.5406 is the X-ray wavelength for Cu Kα, Å, and θ is Bragg’s diffraction angle, rad.

The average linear size of crystallites *L*_c_ and *L*_a_ was determined in Å according to the Scherrer and Warren equations^32,33^
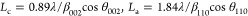
2

where 0.89 is the Scherrer constant that is conditionally equal for cokes to ensure uniformity of the published results,^34^ 1.84 is the constant derived by Warren for two-dimensional particle size,^33^ and β is the diffraction line width at the maximum half-height (rad) exclusive of the instrumental peak width *b* = 0.2°.

#### 2.2.4 Thermogravimetric and Differential Thermal Analyses

The thermogravimetric and differential thermal analyses (TGA–DTA) were conducted using an SDT Q600 thermal analyzer. The mass of the petroleum coke additive sample was taken within the range of 8.158–12.641 mg. The heating was conducted from 50 to 900 °C at 20 °C/min. The oxidizing atmosphere used was air.^35^

## 3 Results and Discussion

The material balance of the delayed coking process of the heavy petroleum residues with the produced petroleum coke additive is given in Table 5.

**Table 5:** Material Balance for Producing the Petroleum Coking Additive

material balance	concentration, wt %		
input			
vacuum residue (VR1)	100.00		
vacuum residue (VR2)		100.00	
visbreaker residue (VR3)			100.00
output			
the amount of distillates, including	51.97	50.20	46.83
gasoline (IBP–180 °C)	18.70	16.53	12.84
light gasoil (180–360 °C)	31.77	31.35	30.97
heavy gasoil (360 °C–FBP)	1.50	2.32	3.02
petroleum coking additives	33.86	34.82	39.29
hydrocarbon gas + losses	14.17	14.98	13.89
total	100.00	100.00	100.00

The maximum yield of the petroleum coking additive was obtained in the process of coking the VR3 visbreaking residue and amounted to 39.29%. This is due to the presence of a larger number of reactive molecules in the visbreaking residue, compared to vacuum residues. The yield of the petroleum coking additive during vacuum residue coking was 33.86% for VR1 and 34.82% for VR2. The distillate yield is reduced in a reverse direction from 51.97% for VR1 to 46.83% for VR3. The produced distillates that have been subjected to hydroremoval of sulfur-containing compounds can be used as the motor fuel components.

The quality indicators of the petroleum coking additive were determined from the point of view of its further partial use instead of the coking coals and are given in Table 6.

**Table 6:** Quality Indicators of the Petroleum Coking Additive

properties	VR1	VR2	VR3
moisture content (*W*^a^), %	0.150	0.520	0.528
volatile-matter yield (*V*^daf^), %	17.32	19.07	16.15
microstructure score	3.1	2.8	3.0
ash content (*A*^a^), %	0.15	0.23	0.20
real density (*d*_r_), g/cm^3^ (without calcination)	1.230	1.392	1.528
apparent density (*d*_a_), g/cm^3^	0.956	1.065	1.424
total porosity, %	22.3	23.0	7.0
sulfur content, wt %	3.46	2.98	2.81
microhardness, N/mm^2^ (max. load 49–50 mN)	51.8	56.7	49.7

The least volatile-matter yield (16.15%) from the samples produced in the course of experiments is normally characteristic of the petroleum coking additive VR3, as crude petroleum undergoes mild thermal cracking before its production. The real density and apparent density for the VR1 and VR2 correlate with the volatile-matter yield. The low porosity of the VR3 sample as compared to the VR1 and VR2 sets a higher density value. The sulfur content in the produced samples ranges from 2.81 to 3.46%.

**Figure 3:**
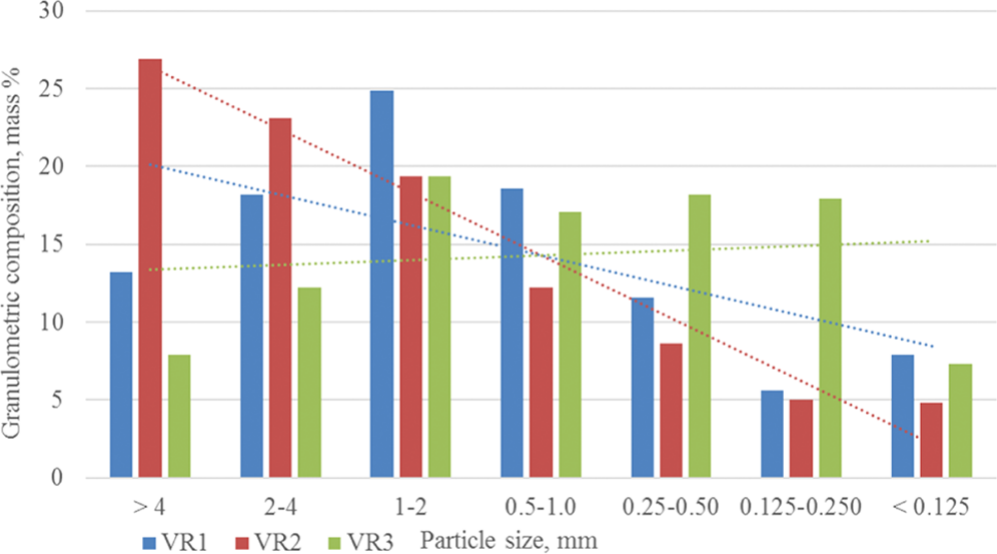
Particle size of the petroleum coking additive produced from coking of three kinds of crude materials: VR1, VR2, and VR3.

The particle size of the petroleum coking additive produced from coking of three kinds of crude materials and after its mechanical removal from the reactor is shown in Figure 3.

The results demonstrate that VR1 and VR2 petroleum coking additives have a larger number of coarse particles than the additive from VR3. This points to the higher mechanical strength of the petroleum coking additive produced from vacuum processing of products of the heavy petroleum residues than that of the carbon material produced from the product of the thermal cracking. These conclusions are also confirmed by the microhardness determination results. With a maximum load of 49–50 mH, the VR3 petroleum coking additive has the least microhardness that increases when switching from VR1 raw materials to VR2.

The structure of the petroleum coking additive is amorphous carbon. The production temperature of the petroleum coking additive is 40–50 °C lower than that of the petroleum coke while having equal coking time. In the case of high-temperature treatment, the amorphous carbon transforms into graphite. The crystallites formed in the amorphous matrix of the petroleum coking additive have a turbostratic structure that is different from the graphite structure during thermolysis.^36^ The main structural parameters of such crystals are the interplane distances *d*_002_ and *d*_110_ and the coherent scattering area *L*_c_ and *L*_a_ in the directions of crystal axes “c” and “a”, respectively. For graphite, *d*_002_ and *d*_110_ are 3.354 and 1.232 Å, respectively, and for the turbostratic structure, *d*_002_ changes within 3.37 and 3.60 Å, and *d*_110_ changes within 1.215–1.230 Å.^37^

**Figure 4:**
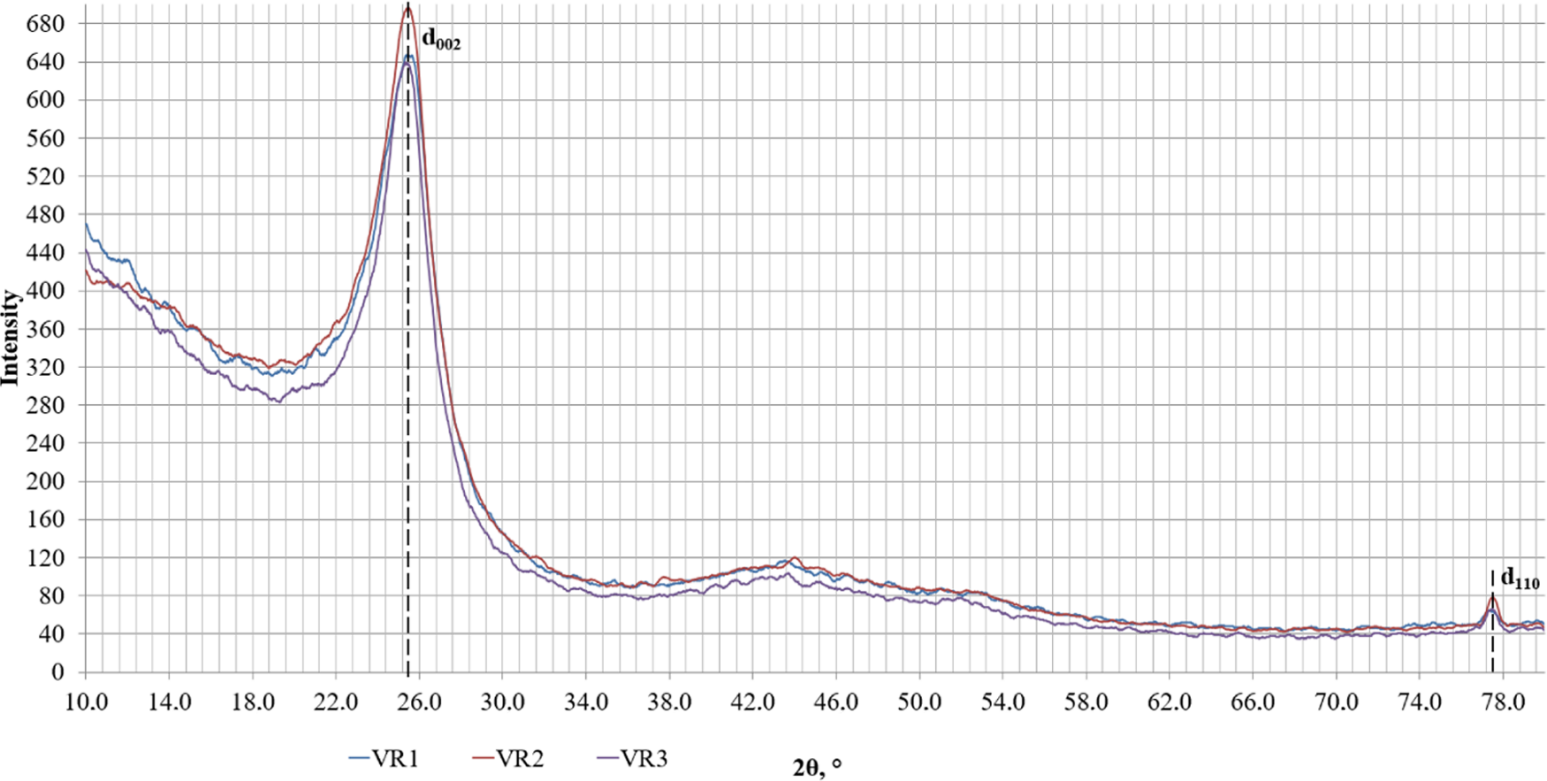
Comparison of X-ray diffraction patterns of the petroleum coking additive produced from coking of three kinds of crude materials: VR1, VR2, and VR3.

Figure 4 shows the comparison of X-ray diffraction patterns of the petroleum coking additive produced from three kinds of heavy petroleum residues.

The peaks with maximum 2θ angles of 25 and 77° that are correspondingly responsible for reflections 2θ_002_ and 2θ_110_, respectively, remain apparent. The angular position of the reflections (2θ_002_ and 2θ_110_) on the X-ray diffraction pattern is determined by the corresponding interplanar distance (*d*_002_ and *d*_110_).^38,39^ The results of the diffraction analysis and design values by reflections (002) and (110) for the petroleum coking additives produced from coking of three kinds of crude materials VR1, VR2, and VR3 are given in Table 7.

**Table 7:** Diffraction Analysis Results of the Petroleum Coking Additive

	estimate from reflection (002)				estimate from reflection (110)			
no.	2θ, deg	FWHM, deg	*d*_002_, Å	*L***_c_**, Å	2θ, deg	FWHM, deg	*d*_110_, Å	*L*_a_, Å
VR1	25.360	7.5800	3.5092	10.626	77.380	0.9800	1.2323	216.893
VR2	25.420	5.7200	3.5011	14.087	77.500	0.7000	1.2307	310.451
VR3	25.320	4.8400	3.5147	16.650	77.480	0.7000	1.2309	310.408

The interplane distance *d*_002_ increases from 3.5092 to 3.5147 Å with increasing heaviness of the crude material from VR1 to VR3, and *d*_110_, by contrast, decreases from 1.2323 to 1.2309 Å. According to the X-ray diffraction analysis results, the microstructure of the petroleum cokes can be judged on the basis of the ratio of the average height *L*_c_ and average diameter *L*_a_ of crystallites.^40,41^ In this case, the *L*_c_ to *L*_a_ ratio is about 1–10 that speaks for the flattened structure of crystallites and is caused by thermobaric conditions for the petroleum coking additive formation.

**Figure 5:**
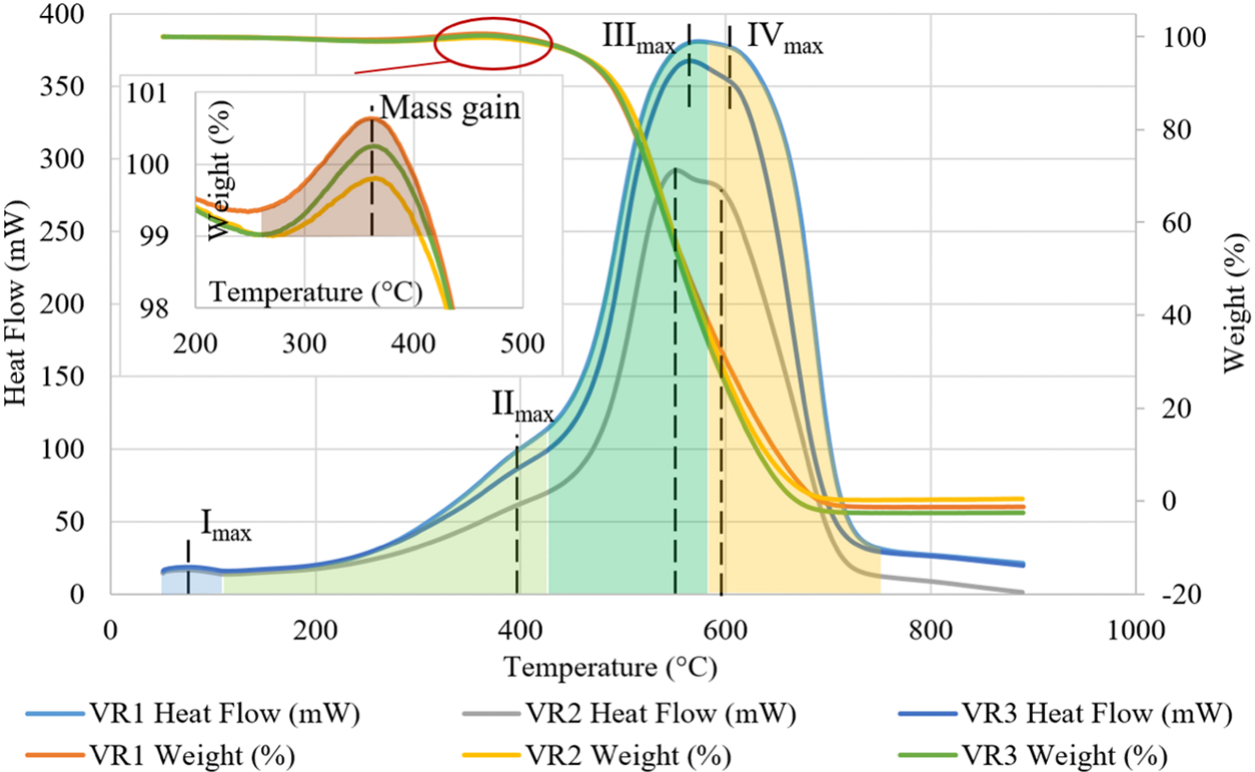
Thermogravimetric and differential thermal analyses of the petroleum coking additive produced from coking of three kinds of crude materials: VR1, VR2, and VR3.

Thermogravimetric and differential thermal analyses were conducted for all petroleum coking additive samples to assess chemical transformations that occurred during their further high-temperature treatment in the charge, like in similar studies on carbon materials.^42^ The thermogravimetric and differential thermal analysis results of the VR1, VR2, and VR3 petroleum coking additives are shown in Figure 5.

All of the tested temperature ranges for the petroleum coking additive samples can be divided into four regions with maximum endothermal effects that generally coincide for all three samples of the carbon materials.

The first zone with a maximum (I_max_) of 75–80 °C is within the range from 50 to 100 °C and characterizes the moisture removal from the carbon material samples. The second zone with a maximum (II_max_) of about 400 °C is within the range from 100 to 439–445 °C and is associated with the mass increase to 0.6% of the sample initial mass. This mass increase is likely due to the sulfur oxidation to SO_2_/SO_3_ and further absorption of the part of these sulfurous gases by the other microelements contained in the petroleum coking additive, with the formation of the relevant sulfates (that is NiSO_4_, CaSO_4_, FeSO_4_, etc.).^43^ When the temperature increases above 400 °C, the petroleum coking additive starts burning when in contact with the ambient oxygen and quickly loses its mass, while the carbonization simultaneously occurs. The third zone with a maximum (III_max_) of about 550–560 °C is within the range from 439–445 to 570–580 °C and is caused by the volatile-matter yield during the petroleum coking additive burning. The fourth zone with a maximum (IV_max_) of about 594–605 °C is within the range from 570–580 to 737–740 °C and is characteristic of carbon burning. However, with such a high heating rate as 20 °C/min, two zones with III_max_ and IV_max_ form a single peak in general. The sample was subjected to heavy thermal exposure only at the stage of the volatile-matter yield, and the generated heat cannot reach the sample internal surface in time. The lacking heat leads to the fact that the sample shows a less intensive reaction at the stage of carbon burning.^44^ At a temperature above 740 °C, almost all burning reactions are completed, and the residual sample mass includes the ash content of the petroleum coking additive.

## 4 Conclusions

Obtaining a petroleum coking additive as a partial replacement for coking coals (for obtaining a charge in the metallurgical coke production) can be performed in delayed coking units of oil refineries as an alternative to obtaining lumpy petroleum coke.

The petroleum coke is mainly used as the crude material for production of electrodes and anode paste and for preparation of regenerating graphitized carbon materials at the iron and steel production units. Strict requirements are specified for their sulfur content: 0.5–1.0 wt %. When using sulfurous heavy petroleum residues, it is impossible to obtain low-sulfur petroleum coke only by delayed coking; it is necessary to use additional desulfurization processes, which are not always economically viable.

In the course of experimental studies, from heavy petroleum residues of Ltd Kinef (Russia), two types of vacuum residue and visbreaking residue with a sulfur content from 2.81 to 3.15% were obtained; in addition to a large amount of distillates (46.83–51.97%), a carbon material was also obtained with a yield of volatile substances from 16.15 to 19.07% in amounts of 33.86–39.29%. It is amorphous carbon with characteristic reflections of (002) and (110) and a thermogram, which is a petroleum coking additive and can be used to partially replace coking coals in the charge in metallurgical coke production.
